# Immune Checkpoint Inhibitor Therapy for Kidney Transplant Recipients – A Review of Potential Complications and Management Strategies

**DOI:** 10.3389/ti.2024.13322

**Published:** 2024-10-16

**Authors:** Elena Bianca Barbir, Samer Abdulmoneim, Arkadiusz Z. Dudek, Aleksandra Kukla

**Affiliations:** ^1^ Division of Nephrology and Hypertension, Mayo Clinic, Rochester, MN, United States; ^2^ Department of Surgery, Mayo Clinic, Rochester, MN, United States; ^3^ Department of Medical Oncology, Mayo Clinic, Rochester, MN, United States

**Keywords:** immunology, rejection risk, oncology, kidney transplant, biomarkers, immunotherapy

## Abstract

Immune checkpoint inhibitor (ICI) therapy has enabled a paradigm shift in Oncology, with the treatment of metastatic cancer in certain tumor types becoming akin to the treatment of chronic disease. Kidney transplant recipients (KTR) are at increased risk of developing cancer compared to the general population. Historically, KTR were excluded from ICI clinical trials due to concern for allograft rejection and decreased anti-tumor efficacy. While early post-marketing data revealed an allograft rejection risk of 40%–50%, 2 recent small prospective trials have demonstrated lower rates of rejection of 0%–12%, suggesting that maintenance immunosuppression modification prior to ICI start modulates rejection risk. Moreover, objective response rates induced by ICI for the treatment of advanced or metastatic skin cancer, the most common malignancy in KTR, have been comparable to those achieved by immune intact patients. Non-invasive biomarkers may have a role in risk-stratifying patients before starting ICI, and monitoring for rejection, though allograft biopsy is required to confirm diagnosis. This clinically focused review summarizes current knowledge on complications of ICI use in KTR, including their mechanism, risk mitigation strategies, non-invasive biomarker use, approaches to treatment of rejection, and suggestions for future directions in research.

## Introduction

**FIGURE 1:**
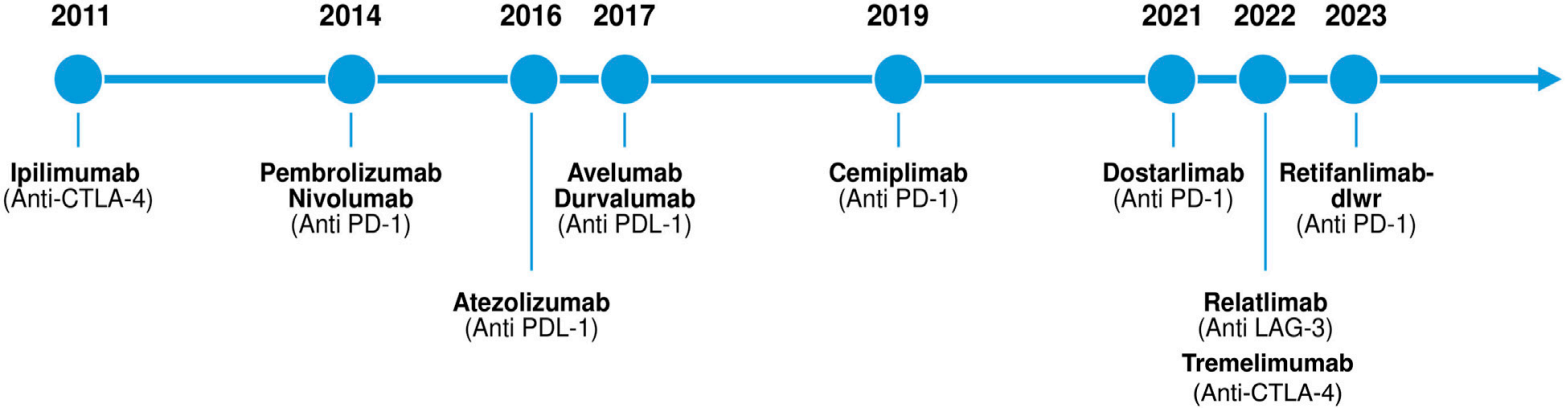
Timeline of immune checkpoint inhibitor approvals by the United States Food and Drug Administration (FDA) from 2011 to 2023.

The last decade has seen a paradigm shift in Oncology with the initial United States Food and Drug Administration approval of immune checkpoint inhibitor (ICI) therapy in 2011 with Ipilimumab. There are currently 10 ICI agents approved (Figure 1) with indications in over 85 malignancies [1]. The momentum has been sustained by the demonstrated efficacy of these agents in the treatment of certain aggressive cancers [2]. Post-transplant malignancy represents a leading cause of death with a functional allograft in kidney transplant recipients (KTR) after the first year post transplant [3]. Prior to the era of immunotherapy, there was no significant improvement in cancer related outcomes over three decades [3, 4]. Due to concerns of attenuated anti-tumor responses and increased risk of toxicity related to allograft rejection, KTR were historically excluded from ICI clinical trials. Early retrospective data affirmed initial concerns with kidney allograft rejection rates as high as 40%–50%. More recently, small prospective trials have reported lower rates of 0%–12% [5–7]. This significant discrepancy in outcomes between the early retrospective and recent prospective data has highlighted the need for additional prospective studies to help guide decision making around maintenance immunosuppression for these patients. Although there is no definitive data on frequency and grade of immune related adverse events (irAEs), including recurrent glomerulonephritis (GN), retrospective data suggest a decreased frequency of irAEs in KTR [5, 8]. The initial hypothesis that immunotherapy is less effective in immunosuppressed solid organ transplant recipients (SOTR) has been challenged by the accruing data; most recently by results from two small prospective trials that reported objective response rates of about 50% in KTR – similar response rates as seen in the general population [6, 7, 9–12]. This review aims to highlight current knowledge around the risks associated with ICI therapy use in KTR, including their mechanism, risk mitigation strategies, the role of non-invasive biomarkers as well as our proposed approach to the management of these patients.

## Immune Checkpoint Inhibitor Therapy Mechanism of Action

**FIGURE 2:**
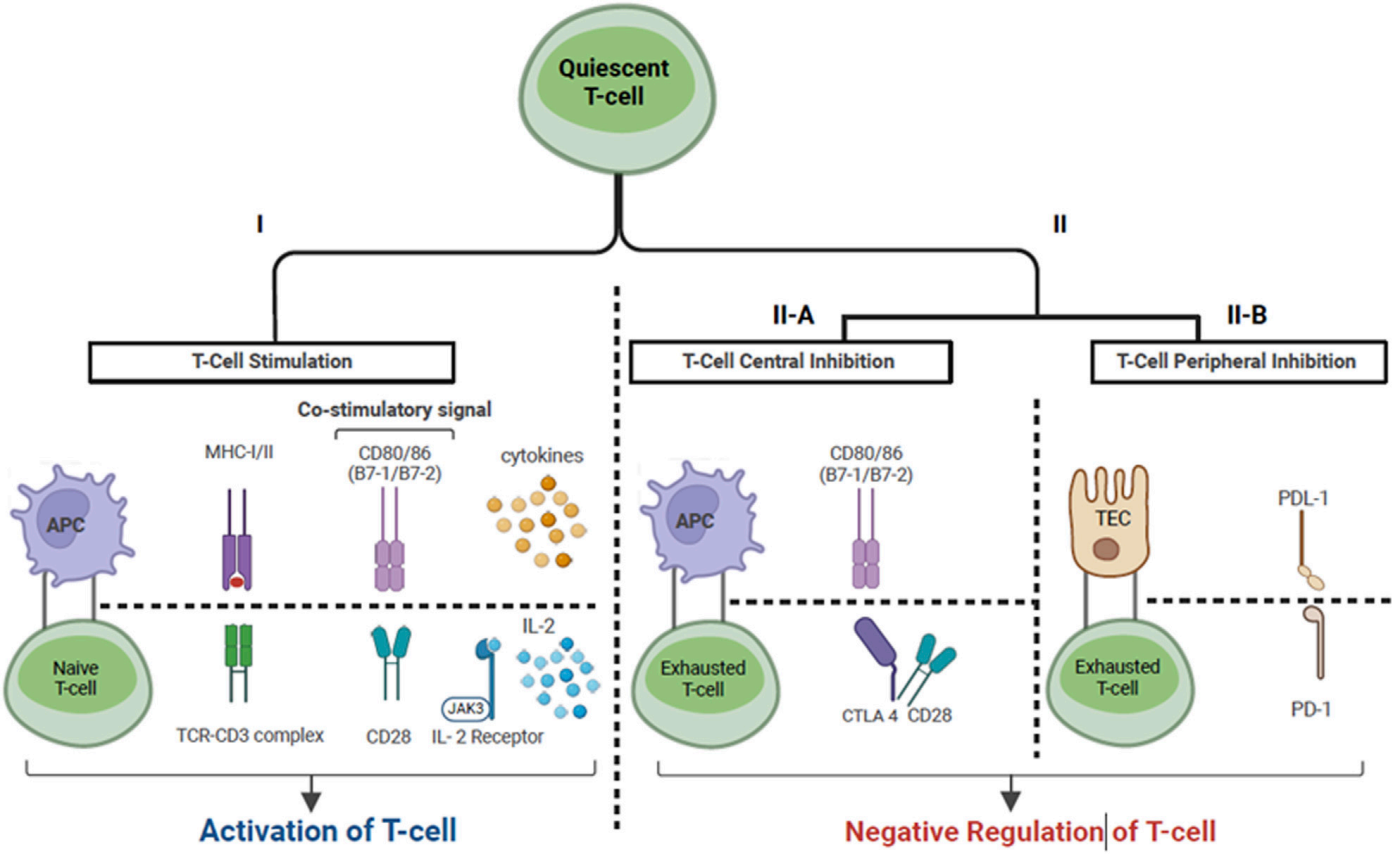
T-cell activation via the three-signal model. Cancer cells express tumor associated antigens which are captured by APCs. Signal 1: Antigen peptide presented by APC on MHC molecule binds to TC on -cell surface. Signal 2: Co-stimulation. The binding of CD28, expressed on T cells, to CD80/86 expressed on APC’s describes one of the necessary co- stimulatory signals. Signal 3: Once signal 1 and 2 have been completed, signal 3 denotes cytokine production by T cells, which allows ongoing T cell differentiation and proliferation. This includes the production of IL2 by T-cells leading to IL2-R stimulation on the surface of T cells. Il- Immune checkpoints provide a negative feedback mechanism in the setting of T cell activation. In lymphoid tissues, CTLA-4 binds to CD80/86 with higher affinity than CD28, leading to competitive inhibition of signal 2. In the peripheral tissue, PD-L1 which is expressed by epithelial cells (i.e., renal TEC, tumor cells) binds to PD-1, which is expressed by peripherally circulating T cells, inducing T-cell exhaustion. Abbreviations: APC, Antigen presenting cell; MHC, Major histocompatibility complex I/ll; TC, T-cell receptor; IL-2, Interleukin-2; IL2-R, Interleukin-2 receptor; CTLA-4, Cytotoxic -lymphocyte associated protein 4; PD-L1, Programmed cell death ligand 1; PD1, Programmed cell death protein 1; TEC, Tubular epithelial cell; JAK-3, Janus Kinase 3.

**FIGURE 3:**
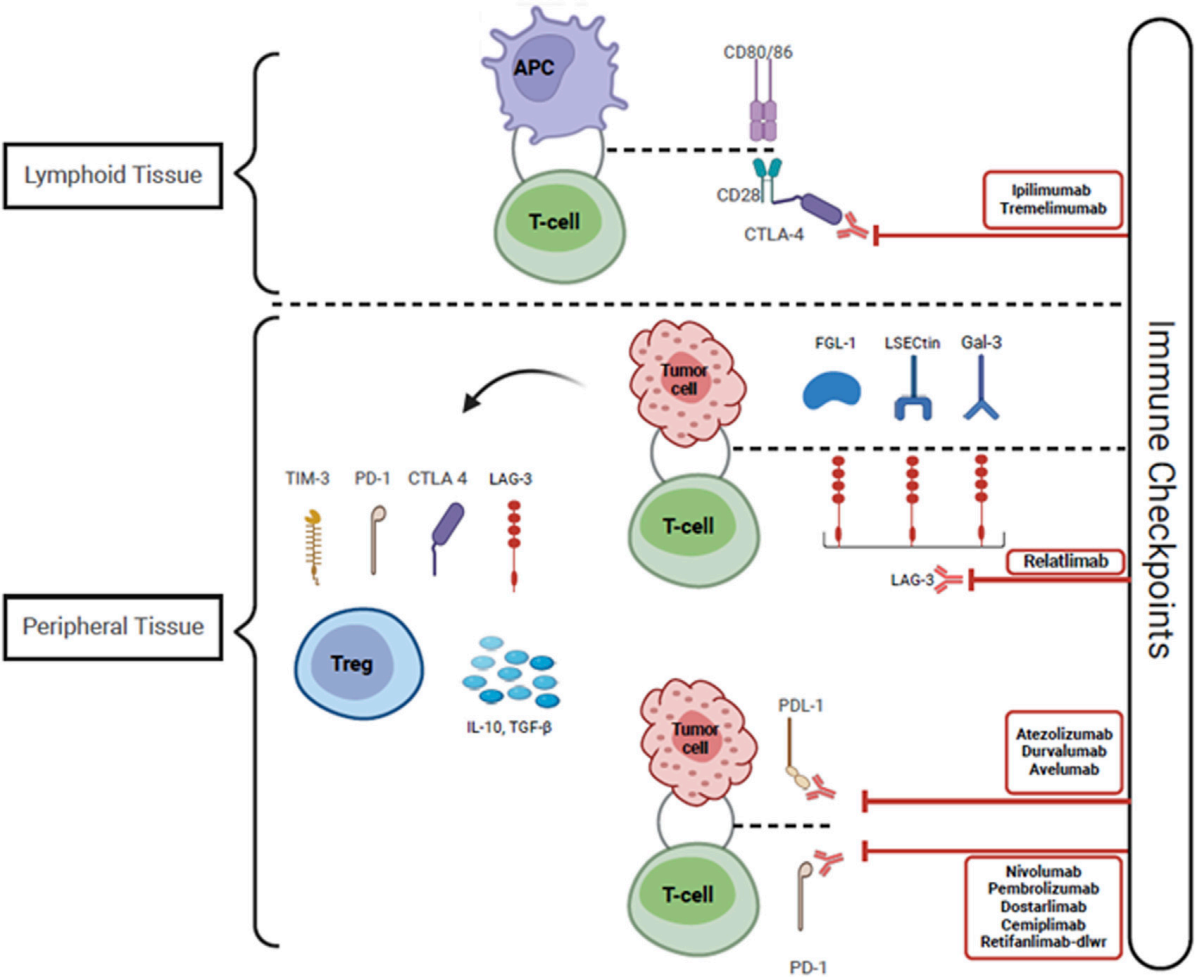
Immune checkpoint inhibitors including Anti CTLA-4, PDL-1 and PD-1 antibodies function by blocking the interactions between checkpoint proteins and their receptors. This disrupts the counter-regulatory negative feedback mechanism that suppresses T-cell activity, leading to persistent T-cell activation and proliferation, allowing T-cells to recognize and eliminate tumor cells. The tumor cells express LECtin, Gal-3, and FGL-1, which bind to LAG-3 expressed on the surface of T-cells and provoke T-cell anergy. Tumor tolerance is also promoted by the infiltration of Tregs within the tumor microenvironment. These Tregs express higher levels of CTLA-4, PD-1, LAG-3, and TIM-3, and they secrete elevated levels of IL-10 and TGF-ß, thus facilitating tumoral resistance. Abbreviations: CTLA-4, Cytotoxic T-Lymphocyte Antigen 4; PDL-1, Programmed cell death ligand 1; PD-1, Programmed cell death protein 1; LSECtin, Liver sinusoidal endothelial cell lectin; Gal-3, Galectin-3; FGL-1, Fibrinogen-like protein-1; LAG-3, Lymphocyte activation gene-3; Treg, Regulatory T-cell; TIM-3, T-cell immunoglobulin and mucin domain-containing protein 3; TGF-ß, Transforming growth factor- beta; CD 80/86 and CD 28, Cluster of differentiation 80/86 and 28; IL-10, Interleukin-10.

Cancer immunotherapy as a category encompasses all therapies whose anti-tumor mechanism is exerted via the activation and expansion of the host immune response to tumor antigens [13]. Specifically, ICI’s enable amplified tumor-reactive T cell responses by disabling intrinsic attenuation mechanisms which lead to T cell exhaustion. Under normal physiologic conditions “immune checkpoints” exist to regulate T cell responses and prevent excessive activation. However, T cells infiltrating the tumor microenvironment are subject to over-attenuation due to tumor immune escape, allowing tumor cells to evade the host immune response [14]. One of the mechanisms of tumor immune escape is the constitutive expression of immune checkpoint ligands, such as programmed cell death ligand 1 (PDL-1) on tumor cells [14]. This allows peripherally circulating T cells expressing programmed cell death protein 1 (PD1) to bind to PDL-1 and become anergic. The PD1/PDL-1 immune checkpoint pathway provides a mechanism for T cells to recognize “self”, as multiple host cells express PDL-1 [15]. Broadly speaking, ICIs are monoclonal antibodies that inhibit immune checkpoint receptors expressed by T-cells from binding to their ligands, and thus enable persistent T cell activation and proliferation. The immune checkpoint pathways that are currently targeted include: 1) the PD1 pathway with its ligands PDL1 and PDL2 which are expressed on lymphoid, myeloid, epithelial cells and tumor cells; 2) the cytotoxic T-lymphocyte antigen 4 (CTLA4) pathway and its ligands CD80/86 which are expressed on myeloid and lymphoid cells, and 3) the lymphocyte-activation gene 3 pathway [15–18]. Figures 2, 3 depict the three-signal model of T cell activation, and how the mechanism of action of ICIs ties into this. This review will focus on the use of PD1/PDL-1 and CTLA4 blockade in KTR as, to our knowledge, LAG3 blockade has not yet been reported in SOTR.

There are multiple hypotheses regarding the mechanism by which ICI use can trigger allograft rejection. Pre-clinical studies using murine and porcine models exist which identify the PD1/PDL-1 pathway as a mechanism of peripheral tolerance, with its disruption linked to auto-immunity and alloreactivity [19–23]. Other potential mechanisms include activation of quiescent alloreactive and effector memory T cells with ICI use illustrated by Dunlap et al; tumor and allograft antigen homology leading to the formation of cross-reactive T-cells as has been demonstrated in 2 cases of myocarditis but yet to be demonstrated in SOTR; and functional inhibition of regulatory T-cells via CTLA-4 and PD-1 inhibition [24–26].

## Incidence of Allograft Rejection in Kidney Transplant Recipients – A Historical Perspective

Our understanding regarding risk of allograft rejection has been evolving. Initially, rejection risk was reported as 40%–50%. This estimation was based on the results of retrospective studies published up until 2021 [5, 8]. Significant reduction of immunosuppression prior to initiation of ICI was a confounding factor, as demonstrated by Murakami et al.’s multi-center analysis, where 65% of patients underwent changes in maintenance immunosuppression prior to starting ICI, of which 35% had a reduction in the total number of agents [5]. However, discrepancies existed even within early retrospective data with smaller case series reporting rejection in only 15% of patients despite decreased immunosuppression [27, 28]. Due to the overall low biopsy rate in the retrospective studies, patients may have been misdiagnosed with rejection given multiple competing risks for acute kidney injury (AKI). Three prospective studies followed which are presented in Table 1. Carroll et al. enrolled seventeen KTR who were continued on their baseline maintenance immunosuppression through ICI therapy [7]. Two cases of biopsy-proven acute rejection were reported out of seventeen included patients (12%), with one patient suspected to have had pre-existing subclinical rejection based on an elevated urinary chemokine, C-X-C motif chemokine ligand 10 (CXCL10), prior to starting therapy [7]. Subsequently, in the phase 1 CONTRAC-1 trial, twelve KTR with advanced cSCC received the PD-1 inhibitor, cemiplimab, while maintained on a mammalian target of rapamycin inhibitor (mTORi) and prednisone mini-pulse with each treatment cycle [6]. Selection of this maintenance immunosuppressive strategy was based on reports from retrospective studies suggesting a decreased incidence of rejection with preserved anti-tumor activity when using the combination of mTORi and prednisone [5, 30, 31]. Specifically, everolimus or sirolimus was used with a target trough level of 4–6 ng/mL, and the prednisone mini-pulse consisted of prednisone 40 mg day −1 through day 3 of cemiplimab administration, followed by 20 mg daily days 4–6 and then 10 mg daily from day 7 onwards until the next cycle [6]. The study did not exclude patients based on immunologic risk as Carroll et al. had done [7]. They reported no rejection episodes. Lastly, Schenk et al. published the results of a prospective, multi-center phase I/II trial, in which eight evaluable KTR with multiple advanced skin cancers received PD-1 inhibitor monotherapy with nivolumab, and subsequently had the option of transitioning to dual ICI blockade with PD-1 and CTLA-4 inhibition with ipilimumab and nivolumab (6/8) for progressive disease [29]. Maintenance immunosuppression consisted of tacrolimus (trough target 2–5 ng/mL) and prednisone 5 mg daily. Of eight evaluable patients, three experienced biopsy proven allograft rejection (38%); one on ICI monotherapy and two on dual ICI therapy, though the third rejection happened after stopping all treatment, including maintenance immunosuppression [29]. These results suggested that a tacrolimus trough of 2–5 ng/mL and prednisone 5 mg daily may be insufficient to prevent organ rejection.

**TABLE 1:** The 3 published prospective trials to date on the use of immune checkpoint inhibitor therapy in kidney transplant recipients.

Study title	Immune checkpoint inhibitors in kidney transplant recipients [7]	CONTRAC-1 [6]	Nivolumab + tacrolimus + prednisone ± ipilimumab for kidney transplant recipients with advanced Cutaneous cancers [29]
Authors, Year	Carroll et al., 2022	Hanna et al., 2024	Schenk et al., 2024
Patient Number	17	12	8
Tumor Group	Any advanced cancer otherwise meeting ICI indication	Advanced cSCC	Advanced Skin Cancers
ICI Type	16 patients on Anti-PD1 therapy 1 patient on Anti-PDL1 therapy	Cemiplimab (Anti PD1)	Initial Nivolumab (Anti PD1) in 8/8 patients Transition to Nivolumab + Ipilimumab (anti-CTLA4) in 6/8 patients
Maintenance Immunosuppression	Maintain prior baseline maintenance immunosuppression	mTORi and dynamic prednisone taper^a^	Tacrolimus (trough 2–5 ng/mL) and Prednisone 5 mg daily
Rejection	2/17, 11.7%	0/12, 0%	3/8, 37.5%
Allograft Biopsy Findings	2 cases of ACR	N/A	1 case of ACR, Mixed Rejection (ACR + ABMR) × 2
Extra-Renal Immune Related Adverse Events	1/17, colitis	1/12, colitis	2/8, arthralgia, maculopapular rash
Objective Response Rate	53%	45%	25%

^a^ Please see text for dosage details. Abbreviations: ICI, immune checkpoint inhibitor; ACR, acute cellular rejection; cSCC, cutaneous squamous cell carcinoma; mTORi, Mammalian Target of Rapamycin Inhibitor; ABMR, antibody mediated rejection.

## Characteristics of ICI Associated Allograft Rejection

Retrospective data suggest that allograft rejection tends to occur early, with a median time to rejection of 3–4 weeks, and is treatment refractory in 50%–80% of patients [5, 8, 32]. However, in a recent multi-center retrospective study including 30 KTR and 1 lung transplant recipient (LTR), Remon et al. noted a comparatively delayed median time to rejection of 8 weeks [33]. This delay may be the result of less aggressive maintenance immunosuppression reduction prior to ICI start.

The data regarding treatment of ICI associated rejection is limited by low sample sizes, and a significant heterogeneity in treatment approaches. Acute cellular rejection (ACR), either alone or in combination with acute antibody mediated rejection (ABMR), has been reported in all biopsied cases to date [5, 7, 8, 29, 32, 34, 35]. For the cases of biopsy proven allograft rejection included the multi-center retrospective study by Murakami et al., 50% consisted of ACR, and 50% consisted of mixed ACR and ABMR, with nine of fourteen biopsied cases with endothelialitis [5]. In the systematic review by Portuguese et al., which included nineteen cases of biopsy-proven rejection, 74% were reported as ACR and 26% as mixed ACR and ABMR [8]. As the 3 available prospective studies to date have small numbers of patients, with only a few reported episodes of allograft rejections, the data is mixed (Table 1).

## ICI Associated Allograft Rejection – Risk Factors

The data we have to date suggests that significant reduction in baseline immunosuppression is a risk factor for ICI associated allograft rejection though the ideal degree of immunosuppression remains to be defined [5, 8, 36]. Prospective evidence suggests that maintaining patients’ prior baseline immunosuppression, or using a dynamic steroid and mTORi reduces the risk of rejection [6–8]. However, despite the encouraging objective response rate noted in these small studies, there remains the concern that maintaining higher degrees of maintenance immunosuppression may blunt the anti-tumor efficacy of ICI. Specifically, high dose steroids have been associated with decreased progression free survival in non-transplant patients with non-small cell lung cancer (NSCLC) [37]. To definitively answer this question, prospective studies comparing allograft and cancer outcomes with different immunosuppressive strategies are needed.

Other risk factors for ICI associated allograft rejection suggested by retrospective data include a prior history of allograft rejection, anti-PD1 therapy or dual ICI therapy, and low dose corticosteroids (<10 mg per day) [5, 8, 24, 31, 35, 36, 38, 39]. Notably, the prospective studies reported by Carroll et al. and Hanna et al. did not exclude patients with a prior history of rejection, and yet low rejection rates were seen [6, 7]. However, Carroll et al. did account for immunologic risk in a different fashion by excluding patients with a donor specific antibody mean fluorescence intensity (DSA MFI) greater than 4,000 [7]. Schenk et al. excluded all patients with any existing DSA or a history of allograft rejection within 3 months prior to enrollment and noted a higher rejection rate [29]. To date, no clear relationship between cancer type and risk of allograft rejection has been established; adequately powered studies are needed to address this question.

## ICI Associated Allograft Rejection – Optimizing Immunosuppression

An immunosuppressive strategy with at least 2 agents and a prednisone dose greater than or equal to 10 mg daily is supported by the current body of evidence. The use of mTORi as maintenance immunosuppression has been associated with a decreased rejection risk in retrospective studies, and further supported by the absence of rejection in the CONTRAC-1 study over a median follow up period of 6.8 months, though those patients were on higher doses of prednisone [5, 8, 27, 38]. There are clinical scenarios in which transition to mTORi is either not tolerated by patients due to drug-related toxicities, or not feasible due to the presence of: 1) healing wounds 2) proteinuria with a urine protein to creatinine ratio >0.5 g/g, or 3) high immunologic risk especially within the first 6 months post-transplant [40–42]. In these situations, there is minimal data to guide decision making. The available data would suggest continuing patients on their prior maintenance immunosuppression, as per Carroll et al. [7] Alternatively, pursuing dual maintenance immunosuppression with prednisone 10 mg daily, and tacrolimus with a trough level 5–7 ng/mL can be considered [29]. Two small, single center retrospective studies demonstrated low rates of rejection with tacrolimus use, either as monotherapy or dual therapy with corticosteroids; when available, the reported achieved tacrolimus trough levels were greater than 4 ng/mL [27, 28]. It is also notable that 70% of patients included in Carroll et al.’s study had maintenance immunosuppressive regimens containing a calcineurin inhibitor (CNI) [7]. Moreover, three prior reviews demonstrated a protective effect with CNI use, though the data analysis was done for all SOTR and not KTR alone [8, 35, 36].

## Allograft Rejection - Treatment

Treatment would ideally be targeted to the histopathologic lesion identified on allograft biopsy. Multiple different approaches to therapy including pulse dose corticosteroids, thymoglobulin, intravenous immunoglobulins, infliximab and plasma exchange to remove circulating ICI have been reported, though no specific treatment approach has consistently demonstrated improved allograft outcomes [5, 7, 29, 35]. The use of lymphodepleting therapies in the treatment of allograft rejection requires careful consideration in the setting of active, advanced malignancies. Allograft irradiation has been trialed for patients with treatment-refractory rejection with limited responses though this may be an option for KTR on ICI wanting to avoid additional immunosuppression and risk tumor progression [43]. It is possible that early recognition of allograft dysfunction and prompt initiation of therapy may improve outcomes, though definitive data is lacking. Ultimately, mortality in this patient population has been attributed to malignancy progression, rather than to organ rejection [5, 8].

## Additional Immune Related Adverse Events

The incidence of irAE’s in non-transplant patients on ICI therapy has been reported to be as high as 60%–85% [44, 45]. While irAE can affect any organ system, the most common manifestations in non-transplant patients include rash, arthralgias, endocrinopathies such as hypothyroidism, and colitis [46]. Acute tubulointerstitial nephritis can occur in the native kidney, with an estimated incidence of in 1.4%–3% for patients on ICI monotherapy, and up to 5% on ICI dual therapy, with glomerulopathies seen even less frequently [47–51].

A question that has previously arisen is whether we may be mis-identifying hypersensitivity reactions in the allograft, i.e., acute tubulointerstitial nephritis (ATIN), as T cell mediated rejection (TCMR). Both Banff Grade 1 acute TCMR and ATIN consist of a lymphocyte-predominant tubulointerstitial infiltrate [52]. While gene expression profiling confirmed the presence of significant molecular overlap between ICI-ATIN and ICI-TCMR, the most frequently upregulated transcripts were different suggesting different pathophysiologic mechanisms [53]. The highest frequency expressed genes in ICI-TCMR were associated with interferon signaling, T cell and Natural Killer cell functions, and TNF superfamily members, while in ICI-ATIN they were associated with allergic response components (IgE, mast cells and eosinophils) consistent with hypersensitivity responses [53]. The authors also identified an interferon alpha induced transcript, interferon-alpha inducible protein 27, that could serve as a potential biomarker for ICI-TCMR [53]. Moreover, there exist clinical differences between ICI-ATIN and ICI associated allograft rejection suggesting different underlying mechanisms. Median time to occurrence of ICI-ATIN is reported as 12–16 weeks, as compared to 3–4 weeks for rejection [5, 8, 47, 54–56]. Prior or concurrent extra-renal irAEs have been shown to be associated with an increased risk of ICI-ATIN, but the same association has not been noted for rejection [5, 39, 55]. Additionally, ATIN-associated drugs, such as proton pump inhibitors, nonsteroidal anti-inflammatory drugs, and antibiotics have been associated with an increased risk of developing ICI-ATIN in native kidneys in multiple systematic reviews [57–60]. Conversely, a significant association between ICI associated kidney allograft rejection and ATIN-associated drug use was not seen in the largest retrospective study to date [5].

Interestingly, in SOTR a lower incidence of extra-renal irAEs has been documented. Portuguese et al. identified a 13.4% incidence of extra-renal irAEs in their systematic review, of which pneumonitis was the most common (37.5%), followed by dermatitis (31%), colitis (25%) and hepatitis (12.5%) [8]. Looking at KTR alone a 25% incidence of irAEs was reported in a 69 patient retrospective study, and a systematic review similarly reported a 24.5% incidence [5, 35]. When looking at severe irAEs that lead to ICI discontinuation, a 21% incidence was reported in a multi-center cohort of 31 SOTR, of which 30 were KTR and 1 was a lung transplant recipient [33]. Prospective studies have revealed similarly low incidences (Table 1) [6, 7, 29].

A question that remains unanswered is the risk of recurrent glomerulonephritis (GN) in KTR on ICI therapy. To our knowledge, no publications on the topic exist to date. In reviewing our single center data at Mayo Clinic Rochester, of 21 patients started on ICI therapy, 9 had end stage kidney disease secondary to a glomerulopathy, and of these, one patient experienced recurrent membranous nephropathy which responded to tacrolimus (Table 2) [61].

**TABLE 2:** Description of the immune mediated causes of end stage kidney disease included in Mayo Clinic Rochester’s single center retrospective study on ICI use in KTR.

Number of cases	Glomerulopathy [61]
2	IgA nephropathy
1	IgA vasculitis
1	Primary focal segmental glomerulosclerosis
1	AA Amyloidosis
2	Anti-neutrophil cytoplasmic antibody associated vasculitis
1	PLA2R positive membranous nephropathy
1	Chronic GN of unclear etiology

Abbreviations: IgA, Immunoglobulin A; PLA2R, Phospholipase A2 receptor; GN, glomerulonephritis.

The occurrence of irAEs in the immune-intact population has been correlated with improved anti-tumor efficacy with multiple retrospective studies demonstrating an improved median overall survival, and one study showing an improved objective response rate (ORR) and progression free survival [62, 63]. Presumably, there is a correlation between the amplitude of the tumor-directed T cell response and the off-target occurrence of irAEs. Despite continuation of maintenance immunosuppression in SOTR and an associated decreased incidence of irAEs, the ORR for certain tumor types, such as advanced cutaneous squamous cell carcinoma (cSCC) and melanoma, has been comparable to that seen in the immune intact population [8].

## Tumor Response

**FIGURE 4:**
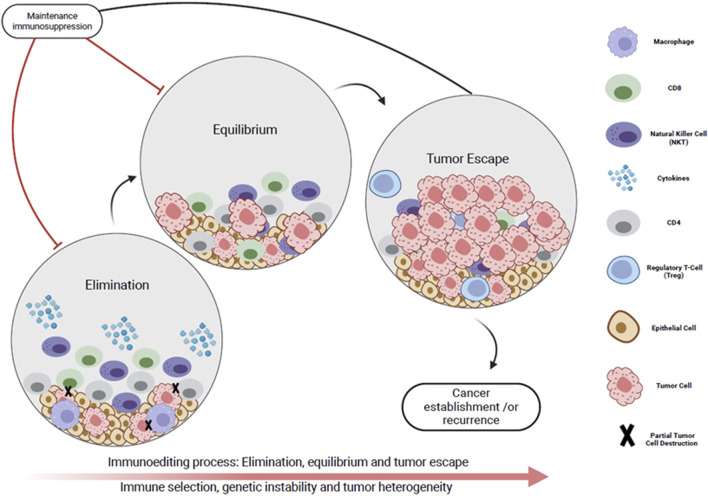
Immunoediting process represented by the dynamic interplay between the tumor micro-environment and the immune system in three phases. Phase I (Elimination): The host immune system initially recognizes the cancer cells as foreign (immune surveillance). Cytotoxic -cells and natural killer cells target tumor cells. Phase I (Equilibrium): A subset of tumor cells develop immune evasion mechanisms (reduced immunogenicity), but there is an overall balance between immune mediated tumor suppression and tumor outgrowth. Phase IIl (Tumor escape): Tumor cells evade the immune system’s surveillance and proliferate uncontrollably. This results in clinically apparent tumor, recurrence and/or metastases. Maintenance immunosuppression impedes initial immune surveillance. This reduces selective pressure on tumor cells which is necessary for the development of mechanisms that allow tumor immune evasion. As a result, tumors that develop in immunosuppressed individuals may be more likely to maintain their initial immunogenicity.

In the CONTRAC-1 study, an ORR of 46% was shown for KTR treated with cemiplimab for advanced cSCC [6]. Portuguese et al. reported an ORR of 68.2% for SOTR receiving ICI for advanced cSCC [8]. By comparison, the reported ORR in the immune-intact population for advanced cSCC is 34%–50% [9]. With regards to cutaneous melanoma, two recent reviews demonstrated a similar ORR in SOTR receiving ICI therapy (ORR 32%–36%) compared with the immune-intact population (ORR on ICI monotherapy 30%–40%, and ORR 61% with dual CTLA4 and PD1 inhibition) [8, 38, 64]. The theory of tumor-immune editing provides a potential explanation for the similar ORR in immunosuppressed and immune intact patients. This refers to the process by which an intact immune system selects for the survival of less immunogenic cancer cells, which subsequently go on to proliferate by evading both the innate and adaptive host immune responses [65]. Tumor cells proliferating in immunocompromised hosts may not undergo tumor-immune editing to the same extent, potentially rendering them more responsive to ICI (Figure 4) [66].

Looking at KTR alone, Table 3 highlights data regarding objective response rates for cSCC, melanoma and NSCLC from three recent retrospective studies and one systematic review. This data seems to suggest that KTR have worse ORR when looked at individually, compared to ORR data for all SOTR analyzed cumulatively. Currently, prospective data establishing ORR to ICI therapy in kidney transplant recipients with various tumor types is limited, as seen in Table 1.

**TABLE 3:** Objective response rate by tumor group in retrospective cohort studies focusing on kidney transplant recipients alone, and a systematic review.

Study title	A multi-center study on safety and efficacy of immune checkpoint inhibitors in cancer patients with kidney transplant [5]	Immune-checkpoint inhibitors in renal transplanted patients affected by melanoma: A systematic review [67]	Cemiplimab for advanced cutaneous squamous cell carcinoma in kidney transplant recipients [28]	Immune checkpoint blockers in solid organ transplant recipients and cancer: the INNOVATED cohort^a^ [33]
Authors, Year	Murakami et al., 2021	Rossi et al., 2021	Van Meerhaeghe et al., 2022	Remon et al., 2024
Patient Sample Size by Tumor Type	cSCC – 24 Melanoma – 22	Melanoma – 32 Uveal Melanoma - 2	cSCC - 7	NSCLC – 11
Immune Checkpoint Inhibitor Regimen by Tumor Type	cSCC Monotherapy: 87.5% Dual Therapy: 12.5% Melanoma Monotherapy: 64% Dual Therapy: 36%	Monotherapy: 100%	Monotherapy: 100%	Monotherapy: 73% Dual Therapy: 27%
Objective Response Rate by Tumor Group	cSCC – 33% Melanoma – 36%	23.5%	42.8%	45.5%

^a^ The NSCLC sub-group of the INNOVATED cohort consisted of KTR alone. Abbreviations: cSCC, cutaneous squamous cell carcinoma; NSCLC, Non-Small Cell Lung Cancer.

Special considerations with regards to tumor response exist. A literature review which included 94 KTR on ICI found that those with preserved allograft function maintained on CNI have worse tumor response rates than those maintained on mTORi, emphasizing the benefit of a transition to mTORi whenever feasible [38]. This association is suggested in Schenk et al.’s study in which an ORR of 25% was reported despite a low degree of maintenance immunosuppression, suggesting that the use of tacrolimus may impede ICI anti-tumor efficacy [29]. This finding deserves further study as it remains unclear if the difference in outcome is due to: 1) the CNI blunting ICI anti-tumor efficacy, 2) patient selection factors, or 3) the intrinsic anti-neoplastic activity of mTORi. Furthermore, it has not been established if this effect is dependent on tumor type [66]. There is mechanistic evidence suggesting that mTORi can promote the maintenance of the anti-tumor effects of ICI therapy while allowing for the preservation of allograft tolerance [68]. Using peripheral blood immunophenotyping, Esfahani et al. demonstrated that concurrent administration of anti-PD1 therapy and mTORi in a KTR with melanoma led to tolerogenic changes including suppression of global T cell activation and preservation of the regulatory T cell population, while maintaining the circulating levels of a subset of tumor directed T cells (interferon gamma producing CD4^+^ T cells and cytotoxic CD8^+^ T cells) [68].

## Future Considerations

Additional risk stratification tools would help guide decision making around maintenance immunosuppression optimization prior to ICI initiation. To this end, several biomarkers have been proposed which have not yet been widely clinically validated. A systematic review identified a correlation between positive PDL-1 allograft staining in liver transplant recipients (LTR) and one KTR and ICI associated rejection [8]. All LTR with positive PDL-1 staining experienced rejection (n = 6), and all those without did not (n = 8) [8]. PDL-1 expression has been shown to represent a tolerogenic mechanism in murine cardiac allograft models [19, 69]. Obtaining protocol renal allograft biopsies and staining them for PDL-1 prior to ICI initiation may help risk-stratify patients and help direct decisions around maintenance immunosuppression. This strategy would also allow for the identification and treatment of sub-clinical rejection prior to ICI start. Notably, the patient with treatment refractory allograft rejection described by Carroll et al. may have been experiencing sub-clinical rejection prior to ICI start [7]. In certain clinical situations, allograft biopsies may pose a higher risk, and center-specific resource limitations may also exist. Non-invasive biomarkers may be used to screen for sub-clinical rejection prior to ICI start and once therapy is initiated. These results could subsequently justify indication biopsies and early therapeutic intervention. Urinary chemokines, C-X-C motif chemokine ligand 9 (CXCL9) and ligand 10 (CXCL10), have both been clinically validated as markers of sub-clinical ACR in KTR who are not on ICI therapy [70–73]. Carroll et al. pre-specified an exploratory endpoint utilizing CXCL10 and noted rising levels in both of the patients who experienced allograft rejection [7, 70]. Another non-invasive biomarker, donor-derived cell-free DNA (dd-cfDNA), has been validated for the detection of renal allograft rejection though not in the context of ICI use [74–76]. Schenk et al. trended dd-cfDNA every 2 weeks in their study, but only noted a clear temporal association between dd-cfDNA elevations and allograft rejection in one of three patients [7]. Additional prospective studies to validate non-invasive biomarkers are needed.

From a therapeutic perspective, there is very limited data on dual ICI use in KTR. A phase 2 prospective trial (NCT05896839) is currently underway to determine tumor response and allograft toxicity in patients with advanced cutaneous cancers on dual ICI therapy with mTORi and prednisone maintenance immunosuppression. Additional future considerations include the use of targeted immunotherapies in KTR with solid organ tumors to reduce the risk of allograft rejection, these include chimeric antigen receptor T-cell therapy (CART) in which T cells are engineered to target tumor specific antigens, or use of oncolytic viruses. Recently, the ARTACUS trial demonstrated encouraging results with a 34.8% ORR for the treatment of advanced cSCC in 27 SOTR with intra-tumoral oncolytic viruses, with no allograft rejections [77].

## Conclusion

Several clear conclusions can be drawn from the existing data: 1) KTR can benefit ICI therapy, 2) KTR are at risk of rejection and treatment related allograft loss while on ICI therapy but this risk can be reduced with optimization of maintenance immunosuppression and potentially with close follow up allowing early intervention, 3) extra-renal irAEs in KTR have been documented less frequently than in the immune-intact population though data on recurrent GN in the allograft is very limited. While we lack high level evidence to direct optimal maintenance immunosuppressive regiments, retrospective data suggests superiority of mTORi over CNI, but no prospective randomized controlled studies comparing the two regiments have been performed. Patients would ideally be risk-stratified prior to ICI therapy initiation. Protocol biopsies and non-invasive biomarkers, such as urine CXCL9, CXCL10 or dd-cfDNA, can be used to screen for sub-clinical rejection. Additional risk stratification with PD-L1 staining of allograft biopsy tissue can be considered. However, all of these interventions require additional clinical validation in the setting of ICI use prior to widespread application. Decisions around timing of ICI therapy initiation, and treatment of allograft complications while on ICI therapy require a patient-centered, multi-disciplinary approach. Transplant centers would benefit from a unified protocol-based approach to the management of KTR with malignancies, co-developed with oncologists. Future research is needed directly comparing different maintenance immunosuppression strategies in a balanced group of patients to help us determine how best to optimize cancer and allograft outcomes.
